# High Nitrogen Supply Induces Physiological Responsiveness to Long Photoperiod in Barley

**DOI:** 10.3389/fpls.2017.00569

**Published:** 2017-04-12

**Authors:** Jian Zeng, Huajin Sheng, Yang Liu, Yao Wang, Yi Wang, Houyang Kang, Xing Fan, Lina Sha, Shu Yuan, Yonghong Zhou

**Affiliations:** ^1^College of Resources, Sichuan Agricultural UniversityWenjiang, China; ^2^Institute of Natural Resources and Geographic Technology, Sichuan Agricultural UniversityWenjiang, China; ^3^Triticeae Research Institute, Sichuan Agricultural UniversityWenjiang, China

**Keywords:** photoperiod, nitrogen supply, physiological response, photosynthesis inhibition, ultrastructural morphology

## Abstract

Photoperiod and nutrient nitrogen (N) supply influence the growth, development, and productivity of crops. This study examined the physiological, biochemical, and morpho-anatomical traits of NA5 and NA9, two barley cultivars with contrasting photoperiod lengths, under the combined treatment of photoperiod regime and N supply. Under long photoperiod, high N supply decreased net photosynthesis; decreased chlorophyll a and chlorophyll a/b; decreased ascorbate peroxidase (APX), catalase (CAT), and superoxide dismutase (SOD) activities; decreased ascorbate, glutathione, soluble protein, and soluble sugar; destroyed mesophyll cell integrity; and increased $O_{2}^{•–}$, malondialdehyde, and proline in both NA5 and NA9. Under short photoperiod, high N content increased net photosynthesis; increased chlorophyll a and chlorophyll a/b; increased APX, CAT, and SOD activities; and increased antioxidants, soluble protein, and soluble sugar in NA9 but decreased the same parameters in NA5. These results indicated that N supply strongly affected photosynthetic capacity and the balance of reactive oxygen species in response to short and long photoperiod. High N supply enhanced the sensitivity of long-day barley to photoperiod change by inhibiting photosynthesis and decreasing antioxidant defense ability. High N mitigated the undesirable effects of shortened photoperiod in short-day barley. Therefore, the data from this study revealed that N status affects adaptation to photoperiod changes by maintaining redox homeostasis and photosynthetic capacity.

## Introduction

The various biological rhythms, such as seed germination, flowering, stem growth, cold acclimation, and dormancy, that alter plant growth and development are controlled by photoperiod via an endogenous oscillator or biological clock to meet day/night cycle requirements (Thomas and Vince-Prue, 1997). Photoperiod length influences biomass production, leaf and cell structure, and chloroplast ultrastructure (Lepistö and Rintamäki, 2012). Short-day plants have thinner leaves with lower chlorophyll a/b ratio and stomatal density than long-day plants; these features are consistent with reports that chloroplast malfunction disturbs the expression of genes that are associated with the circadian clock (Hassidim et al., 2007). The circadian clock regulates photoperiodic development by cooperating with photoreceptor proteins. In numerous plant species, groups of photoperiod-related proteins, including bark storage proteins (BSPs) and vegetative storage proteins (VSPs), temporarily store amino acids to buffer the availability of nitrogen (N) and other nutrients during shoot growth (Paiva et al., 1983; Coleman et al., 1991; Liu et al., 2005; Wildhagen et al., 2013). Photoperiod, antioxidative capacities, and plant growth are likely interrelated given that oxidative damages are prevented by the redirection of redox-mediated acclimation signals, thus allowing more efficient light usage (Becker et al., 2006). By enhancing antioxidant enzyme activities, N fertilization prevents damage to photosynthesis from accumulated reactive oxygen species (ROS) (Medici et al., 2004).

Nitrogen (N) is a major constituent of essential nucleotides and proteins. N is taken up from the soil and utilized for various metabolic processes, including the production of nucleic acids, proteins, and signaling and storage molecules (Robertson and Vitousek, 2009). Increased N supply stimulates the growth, productivity, and photosynthetic capacity of plants by improving enzyme activities in leaves (Bungard et al., 1997; Joel et al., 1997). Nitrate reductase (NR), nitrite reductase (NiR), glutamine synthetase (GS), and glutamate synthase (GOGAT) are the enzymes of N assimilation and metabolism that respond to photoperiod (Stöhr and Mäck, 2001). In *Arabidopsis*, the consequences of shortened photoperiod include high NR, GS, and GOGAT activities and amino acid levels (Gibon et al., 2009). In addition, shortened photoperiod induces lipid peroxidation, membrane deterioration, and ROS accumulation during leaf senescence (Zhao et al., 2009). Apoplastic ROS production is an important response to many biotic and abiotic stress signals and is generated via enhanced enzymatic activities (Gupta and Igamberdiev, 2015). Unfortunately, data on the direct effects of N assimilation on these apoplastic reactions are rare. Jaleel et al. (2009) reported that plants have developed several defense systems in all cellular compartments to counteract the toxic effects of ROS. This defense system includes enzymatic antioxidants, such as superoxide dismutase (SOD), catalases (CAT), and ascorbate peroxidase (APX); SOD and CAT reduce $O_{2}^{•–}$ to H_2_O_2_ and APX decomposes H_2_O_2_, and scavenges ROS (Podgórska et al., 2013). In addition, non-enzymatic antioxidants, such as reduced ascorbate (AsA), glutathione (GSH), and soluble sugar, directly or indirectly induce anti-oxidant defense responses (May et al., 1998; Couée et al., 2006; Foyer and Noctor, 2011).

Barley (*Hordeum vulgare* L.) is a long-day plant and is the fourth major cereal crop that is cultivated worldwide; its transition to reproductive growth is significantly delayed when grown under short photoperiod (Stockinger et al., 2007). The developmental rate of barely is crucially determined by soil nutrient supply, temperature, and photoperiod length. Photoperiod length particularly influences the vegetative growth of barely and plays an important role during spikelet initiation or later reproductive phase (Hay and Kirby, 1991; Slafer and Rawson, 1995; Miralles and Richards, 2000). Altering photoperiod directly alters leaf N content and photosynthetic capability (Comstock and Ehleringer, 1986; Bauerle et al., 2012). As a limiting factor, N supply determines the sensitivity of plants to environmental stress (Pell et al., 1995). Adaptations to photoperiod also involve N and carbon dynamics (Finkelstein and Gibson, 2001). For example, shifting to long photoperiod changes the concentration and composition of soluble sugars and soluble proteins in plant tissues, thus modifying plant growth and development. Clarifying the mechanisms behind the combination of photoperiod/N-regulated physiological and developmental responses is challenging because these factors are part of a complex signaling network with considerable variation between both species and cultivars (Bendevis et al., 2014). The possible links between N supply and photoperiod adaptation have not been investigated in barley cultivars with contrasting photoperiod responses. We hypothesized that, relative to photoperiod shift, (1) N fertilization will increase plant growth and biomass accumulation in two barley cultivars with contrasting photoperiods; and (2) N supply will compensate for the delayed plant growth that is induced by a shortened photoperiod. Therefore, we aimed to investigate and compare the effects of photoperiod on the antioxidant enzyme activities, physiological and biochemical status, and cell ultrastructure morphology of two barley cultivars with contrasting photoperiod responses under two different N supply levels.

## Materials and Methods

### Plant Materials and Experimental Design

Two contrasting photoperiod of barley cultivars, NA5 and NA9, were germinated and grown in growth chambers with temperature and photoperiod control at Institute of Natural Resources and Geographic Technology of Sichuan Agricultural University, Chengdu, China. NA5 is a traditional long day cultivar and NA9 is a short day-length cultivar. NA5 and NA9 possess similar genetic background, which are derived from the hybridization between Zheda9 cultivar from southern China and Ganpi4 cultivar from northern China. Barley seeds were germinated in filter paper wetted with demineralized water for 3–5 days at room temperature of 25°C and then seedlings at three leaves stage were chosen and grown on a continuously aerated modified Hogland’s solution [0.25 mmol L^-1^ phosphorus, 1.25 mmol L^-1^ potassium, 0.25 mmol L^-1^ magnesium, 1.25 mmol L^-1^ calcium, 10 μmol L^-1^ boron, 1 μmol L^-1^ manganese, 0.5 μmol L^-1^ zinc, 0.5 μmol L^-1^ copper, 0.35 μmol L^-1^ molybdenum] in 5 L plastic pot. Nitrogen was added as ammonium nitrate at two concentrations: 0.5 mmol L^-1^ (low N level) and 5 mmol L^-1^ (high N level). The nutrient solution was checked daily to ensure a stable level of around pH 6.5 and replaced every 3 days. The experimental layout was completely randomized with three factorial combinations of two levels of nitrogen, photoperiod, and two genotypes of cultivar, respectively. A total of 120 pots of each cultivar with almost uniform height seedlings were used for different photoperiod treatments. Five seedlings of each cultivar were planted in each pot. All the pots were separately put into six growth chambers (three chambers were set at a photoperiod of 16/8 h day/night, the other chambers were set at a photoperiod of 8/16 h day/night), with 10 pots of each cultivar in each growth chamber. In the parallel experiment, N nutrient was supplied to the seedlings in each growth chamber, at low N level (five pots) and high N level (five pots), separately. Therefore, there have four growth treatments for each cultivar, such as short day with low N (SDLN) treatment, long day with low N (LDLN) treatment, short day with high N (SDHN) treatment, and long day with high N (LDHN) treatment. All treatments were sustained in a controlled condition with a photon flux density of approximately 500 μmol m^-2^ s^-1^. The temperature was 25°C during the day and 15°C during the night, and the relative air humidity was set to a minimum of 60%. In order to compensate for the growth chamber effect, we randomly rearranged the pots in each chamber every 2 weeks during the 56-day experiment, and most of barley plants had 2–3 tillers per plant at end of the treatments.

### Measurements of Pigment and Gas Exchange

At end of the experiment, five plants from each replication were randomly selected to measure dry matter accumulation. Biomass samples were oven-dried (85°C for 48 h) to constant weight and weighed. Chlorophyll a (*Chl* a) and chlorophyll b (*Chl* b) contents were determined according to the method of Lichtenthaler (1987) with slight modifications. Chlorophyll was extracted from 0.2 g fresh leaf using 25 mL, 80% (v/v) chilled acetone for 48 h in the dark, and then measured with spectrophotometer (SHIMADZU UV-1700, Kyoto, Japan). *Chl* a was determined at 663 nm, *Chl* b at 646 nm, and chlorophyll concentration were calculated from equations Chl *a* = 12.21 × A_663_ – 2.81 × A_646_ and Chl *b* = 20.13 × A_646_ – 5.03 × A_663_.

Gas exchange measurements were conducted for the uppermost, fully expanded leaf in five randomly chosen individuals from each treatment between 08:00 and 11:30 am using Li-6400 portable photosynthesis system (LI-COR Inc., Lincoln, NE, USA). Leaves were allowed to equilibrate for at least 5 min at 500 μmol m^-2^s^-1^ photosynthetic photon flux density (PPFD) before the measurements. Leaf temperature was maintained at 25°C and relative humidity at *ca*. 50% inside the cuvette. The CO_2_ concentration in the cuvette during the measurements was 350 ± 5 μmol mol^-1^. Once the steady-state gas exchange rates were observed at these conditions, net photosynthetic rate (*P*_n_), stomatal conductance (*g*_s_), transpiration rate (*E*), and intercellular CO_2_ concentration (*C*_i_) were recorded. The leaves in the chamber were removed after photosynthetic capacity measurements and scanned. Their area was estimated by the ImageJ software. Because the leaves measured for photosynthesis did not fill the chamber window, photosynthetic data were recalculated by considering the actual leaf area enclosed in the chamber.

### Biochemical Traits Investigation

The amount of soluble protein was determined by extraction from frozen leaf samples and quantification using the method of Bradford (1976). About 0.5 g leaves were homogenized in a medium contained 50 mmol L^-1^ phosphate buffer (pH 7.8), 0.1 mmol L^-1^ EDTA, 100 μmol L^-1^ phenylmethanesulfonyl fluoride (PMSF) and 2% (w/v) polyvinylpyrrolidone (PVP). Bovine serum albumin was used as a standard.

Total soluble sugars were detected by the method of Dubois et al. (1956). Briefly, about 0.5 g dried leaves powder in 5 mL, 80% (v/v) ethanol was placed in a water bath at 80°C for 30 min, followed by centrifugation at 20,000 × *g* for 20 min. Samples were then extracted for at least three times, and the supernatants were pooled together and diluted up to 25 mL with deionized water. Soluble sugars content was monitored at 487 nm following the calorimetrical phenol–sulfuric acid method using glucose as the standard.

The production level of $O_{2}^{•–}$ was determined by the absorbance of the product of the hydroxylamine reaction at 530 nm following the description of Wang and Luo (1990). The supernatant of samples extraction (1 mL) was added to 0.9 mL of 65 mmol L^-1^ phosphate buffer saline (pH 7.8) and 0.1 mL, 10 mmol L^-1^ hydroxylammonium chloride. The reaction was maintained at 25°C for 30 min. The 0.5 mL of above reaction mixture was then added to 0.5 mL of 17 mmol L^-1^ sulfonic acid and 0.5 mL of 7.8 mmol L^-1^ α-naphthylamine solutions. After 20 min, 2 mL of ether was added to above solution and mixed well, subsequently centrifuged at 1,500 × *g* at 4°C for 5 min. The pink supernatant was measured at 530 nm with a spectrophotometer (Shimadzu UV-1700, Japan). The absorbance values of mixture were calibrated to a standard curve generated with known concentration of HNO_2_. The $O_{2}^{•–}$ generation rate was calculated as twice the concentration of HNO_2_ using the following formula [$O_{2}^{•–}$] = 2 × [HNO_2_].

The concentration of malondialdehyde (MDA) was measured according to the method of Dhindsa et al. (1981). For each sample, 0.3 g fresh leaf was homogenized in 4 mL of 10% (w/v) trichloroacetic acid (TCA) and centrifuged at 10,000 × *g* for 15 min, after which 1 mL supernatant was mixed with 1 mL of 0.6% (w/v) thiobarbituric acid, heated at 95°C for 30 min and then quickly cooled down on ice. After centrifugation at 10,000 × *g* for 10 min, the absorbance of the supernatant was determined at 532 nm with a spectrometer (Shimadzu UV-1700, Japan) and corrected for no-specific turbidity by subtracting the absorbencies at 600 and 450 nm. The MDA concentration = 6.45 × (A_532_ - A_600_) - 0.56 × A_450_.

Proline (Pro) was measured as described by Singh et al. (1973). About 0.3 g fresh leaves were used to extract Pro with 3 mL of methanol:chloroform:water (12:5:1, v/v/v). The homogenate was centrifuged at 2,500 × *g* for 5 min and the supernatant was recuperate and used for Pro estimation. A volume of 1 mL of supernatant was transferred to a tube and heated in a water bath until methanol evaporation, after which 0.33 mL of ninhydrin solution (0.01 g of ninhydrin, 0.166 mL of 6 mmol L^-1^ sulfuric acid, and 0.25 mL of glacial acetic acid), 0.33 mL of glacial acetic acid and 0.33 mL of water were added to the sample. Then, the tubes were cooled to room temperature, and 2 mL of toluene was added. After 30 s of shaking, two phases were separated, and the absorbance of the upper phase was monitored at 520 nm. Pro was determined by comparison with a 0 to 200 μg Pro standard curve and expressed as μg g^-1^ fresh weight (FW).

Reduced ascorbate (AsA) was determined by the method of Hodges et al. (1996). Briefly, fresh leaves (0.3 g) were homogenized in 5 mL of cold 5% (v/v) *m*-phosphoric acid and centrifuged at 20,000 × *g* for 15 min. About 300 μL of supernatant was incubated for 5 min in 700 μL of 150 mmol L^-1^ KH_2_PO_4_ and 5 mmol L^-1^ EDTA. Color was developed with 400 μL of 10% (w/v) TCA, 400 μL of 44% (v/v) *o*-phosphoric acid, 400 μL of 65 mmol L^-1^ α, α’-dipyridyl in 70% (v/v) ethanol, and 200 μL of 110 mmol L^-1^ FeCl_3_. The reaction mixtures were then incubated at 40°C for 1 h and quantified at 525 nm. A standard curve was developed based on ascorbate in the range of 0–50 μmol L^-1^ in 5% (v/v) *m*-phosphoric acid.

Reduced glutathione was assayed according to Guri (1983) with minor modification. Briefly, a total of 0.3 g leaves was homogenized in ice-cold 5% (w/v) TCA (including 5 mmol L^-1^ EDTA). The homogenate was centrifuged at 10,000 × *g* for 10 min. The reaction mixture contained 0.5 mL of distilled water, 1 mL of leaf homogenate, 1 mL of 0.2 mol L^-1^ potassium phosphate buffer (pH 7.5) and 0.5 mL of the reagent dithiobis-2-nitrobenzoic acid (DTNB). GSH was determined when monitored at 412 nm for 3 min using the spectrophotometer. A standard curve in the range of 0–100 μmol L^-1^ GSH was used.

### Antioxidant Enzyme Activity Assays

About 0.3 g leave samples were homogenized in 5 mL of 50 mmol L^-1^ potassium phosphate buffer (pH 7.8), including 1 mmol L^-1^ EDTA, 1% (w/v) PVP, 0.1 mmol L^-1^ PMSF and 0.2% (v/v) Triton X-100 for the determination of APX, CAT, and SOD activities. The homogenate was centrifuged at 12,000 × *g* for 20 min at 4°C and then used for the enzyme assays.

Ascorbate peroxidase activity was measured using a modification of the procedure of Nakano and Asada (1981). The reaction mixture of a total volume of 3 mL consisted of 50 mmol L^-1^ sodium phosphate buffer (pH 7.8), 0.1 mmol L^-1^ EDTA, 0.1 mmol L^-1^ sodium ascorbate, 2.5 mmol L^-1^ H_2_O_2,_ and 80 μl enzyme extract. The H_2_O_2_-dependent oxidation of ascorbate was followed by a decrease in the absorbance at 290 nm. APX activity was assayed when monitored at 290 nm for 3 min. Activity was based on the rate of oxidized ascorbate production using an extinction coefficient (𝜀 = 2.8 mmol^-1^ cm^-1^). One unit of APX was defined as the amount of enzyme that breaks down 1 μmol of ascorbate min^-1^ g^-1^ of protein.

The CAT activity was assayed by a method described by Aebi (1984). The samples were homogenized and then centrifuged at 12,000 × *g* for 30 min at 4°C. Supernatants were stored at 4°C before analysis. The reaction mixture contained 200 μL of 0.1 mol L^-1^ H_2_O_2_ and 100 μL of cell lysates in 50 mmol L^-1^ phosphate buffer (pH 7.8) in a final volume of 1.0 mL. Samples were incubated for 2 min at 37°C, and the absorbance of the samples was monitored for 5 min at 240 nm using a spectrophotometer. Changes in absorbance were taken to be proportional to the breakdown of H_2_O_2_. The activity was expressed as enzyme units (μmol of H_2_O_2_ decomposed per minute) per gram of FW of leaf samples.

The SOD activity was determined by measuring its ability to inhibit the photochemical reduction of nitroblue tetrazolium chloride (NBT), as described by Giannopolitis and Ries (1977). The reaction mixture with a total volume 3 mL contained 0.3 mL each of 20 μmol L^-1^ riboflavin, 150 mmol L^-1^ methionine and 600 μmol L^-1^ NBT, and 0.1 mL of the extract. The reaction was started with the addition of riboflavin and carried out for 30 min under irradiance of 170 μmol photons m^-2^ s^-1^ provided by a white fluorescent lamp. The absorbance at 560 nm was determined, and the extract volume causing 50% inhibition of NBT reduction was taken as one unit of activity. The specific activity of SOD was expressed as U g^-1^ FW.

### Transmission Electron Microscopy

Leaf segments (1–2 mm in length) were fixed in 3% glutaraldehyde (v/v) in 0.2 mol PBS (sodium phosphate buffer, pH 7.2) for 6–8 h, and post-fixed in 1% osmium tetroxide for 1 h, finally, immersed in 0.2 mol PBS (pH 7.2) for 1–2 h. The leaflets were dehydrated in a graded ethanol series (50, 60, 70, 80, 90, 95, and 100%) and embedded in epon-araldite. Ultra-thin sections (80 nm) were sliced, stained with uranyl acetate and lead citrate, and mounted on copper grids for viewing in the H-600IV TEM (Hitachi, Tokyo, Japan). At least 10 photos for each species were analyzed.

### Statistical Analysis

One-way and three-way analyses of variance (ANOVAS) were performed using the SPSS 19.0 (SPSS Inc., Chicago, IL, USA) for Windows statistical software package. Before ANOVAS, data were checked for normality and the homogeneity of variances, and log-transformed to correct deviations from these assumptions when needed. Individual differences among means were determined by Tukey’s tests of one-way of ANOVAS at a significance level of *p* < 0.05. Three-way of ANOVAS was used to evaluate the effects of genotype, photoperiod, N and their interaction effects.

## Results

### Biomass, Pigment, and Gas Exchange Rate

The plant biomass, *P*_n_, *E, g*_s_, *Chl* a, and *Chl* a/b of NA5 were significant higher than those of NA9 under all treatments except for under SDHN treatment (**Table 1**). Irrespective of short or long day lengths, the *P*_n_, *E, g*_s_, and *Chl* a/b of NA5 were significantly lower under high N condition than those of NA5 under low N condition, whereas the *P*_n_, *E, g*_s_ and *Chl* a/b of NA9 increased under SDHN condition. Moreover, NA9 under LDLN condition exhibited significantly higher plant biomass, *P*_n_, *E, g*_s_, *Chl* a, and *Chl* a/b than NA9 under LDHN treatment (**Table 1**). In addition, *P*_n_, *E, g*_s_, and *Chl* a/b were significantly affected by genotype, photoperiod regime, and N level interactions.

**Table 1:** Biomass, net photosynthesis, transpiration rate, stomatal conductance, intercellular CO_2_ concentration, and pigments of barley plant as affected by photoperiod regime, nitrogen levels, and their combination in barley plants.

Genotype	Day length (h)	Nitrogen supply (mol L^-1^)	Biomass (g plant^-1^ DW)	*P*_n_ (μmol m^-2^ s^-1^)	*E* (mmol m^-2^ s^-1^)	*g*_s_ (mol m^-2^ s^-1^)	*C*_i_ (μmol mol^-1^)	*Chl* a (mg g^-1^)	*Chl* a/b
NA5	8	0.5	3.24 ± 0.13 b	12.07 ± 0.20 b	4.89 ± 0.12 b	0.41 ± 0.02 b	292.67 ± 2.68 ab	0.68 ± 0.02 c	2.32 ± 0.04 b
	16	0.5	4.08 ± 0.19 a	15.33 ± 0.11 a	5.51 ± 0.05 a	0.57 ± 0.04 a	302.94 ± 6.65 a	0.87 ± 0.01 a	3.71 ± 0.09 a
	8	5	2.79 ± 0.04 c	11.38 ± 0.07 c	3.65 ± 0.08 c	0.38 ± 0.01 c	269.54 ± 6.12 cd	0.77 ± 0.02 b	1.90 ± 0.05 d
	16	5	3.24 ± 0.04 b	11.96 ± 0.21 bc	4.85 ± 0.09 b	0.41 ± 0.09 b	295.05 ± 3.46 ab	0.74 ± 0.01 bc	2.19 ± 0.08 bc
NA9	8	0.5	2.51 ± 0.06 d	9.61 ± 0.35 d	2.67 ± 0.09 d	0.29 ± 0.01 d	282.98 ± 5.65 bc	0.62 ± 0.03 cd	1.67 ± 0.02 e
	16	0.5	3.16 ± 0.03 b	12.16 ± 0.15 b	5.06 ± 0.18 b	0.45 ± 0.05 b	297.72 ± 8.16 ab	0.75 ± 0.04 b	2.29 ± 0.02 b
	8	5	3.06 ± 0.03 b	11.42 ± 0.23 b	3.82 ± 0.03 c	0.39 ± 0.02 c	274.24 ± 4.46 c	0.71 ± 0.01 bc	2.29 ± 0.03 b
	16	5	2.54 ± 0.03 d	8.28 ± 0.11 e	2.59 ± 0.15 d	0.22 ± 0.01 d	255.89 ± 4.97 d	0.57 ± 0.01 d	2.13 ± 0.01 c
		*P*: F_G_	^∗∗∗^	^∗∗^	^∗∗∗^	^∗∗^	ns	^∗∗∗^	^∗∗∗^
		*P*: F_D_	^∗∗∗^	^∗∗∗^	^∗∗∗^	^∗∗^	^∗^	^∗∗∗^	^∗∗∗^
		*P*: F_N_	^∗∗∗^	^∗^	^∗∗^	ns	^∗∗^	^∗∗^	^∗∗^
		*P*: F_G × N_	^∗∗∗^	ns	^∗∗^	^∗∗^	ns	^∗∗∗^	^∗∗∗^
		*P*: F_G × D_	^∗∗∗^	^∗^	ns	^∗∗^	^∗∗∗^	^∗∗∗^	^∗∗∗^
		*P*: F_N × D_	^∗^	^∗∗^	^∗∗∗^	^∗∗^	^∗∗∗^	ns	^∗∗^
		*P*: F_G × N × D_	ns	^∗∗∗^	^∗∗^	^∗∗∗^	^∗∗^	ns	^∗∗∗^

*Different letters represent statistical significances between treatment (mean ± SE, *n* = 5) at *p* < 0.05 according to Tukey’s multiple range tests. F_*G*_, genotype effect; F_*D*_, photoperiod regime effect; F_*N*_, nitrogen level effect; F_*G* × *N*_, the interactive effect of genotype and nitrogen level; F_*G* × *D*_, the interactive effect of genotype and photoperiod regime; F_*N* × *D*_, the interactive effect of photoperiod regime and nitrogen level; F_*G* × *N* × *D*_, the interactive effect of genotype, nitrogen level and photoperiod regime. Significant values of the factorial analysis (ANOVA) for the effects are denoted as: ns, no significant; ^∗^*p* < 0.05; ^∗∗^*p* < 0.01; ^∗∗∗^*p* < 0.001.*

### Antioxidant Enzyme Activity and $O_{2}^{•–}$ Generation

**FIGURE 1:**
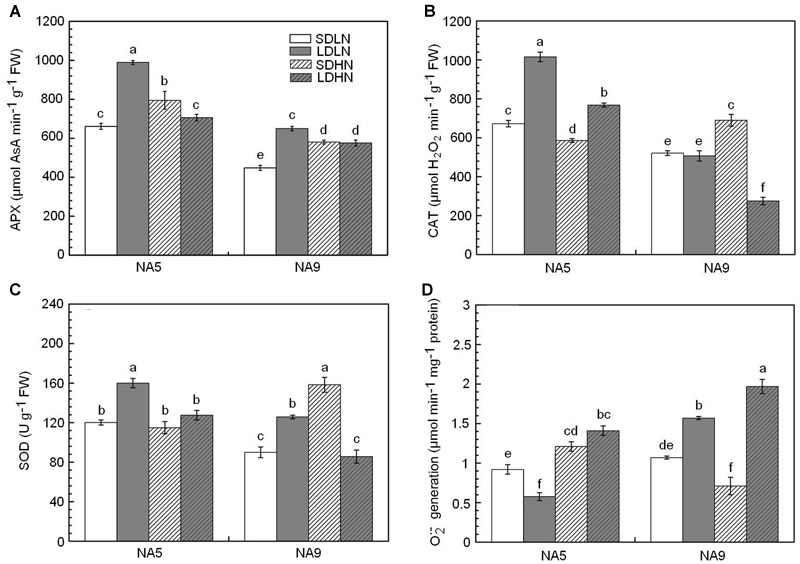
**The activities of ascorbate peroxidase (A)**, catalase **(B)**, superoxide dismutase **(C)**, and germination of $O_{2}^{•–}$ **(D)** of barley plants exposed to the treatments of short day with low nitrogen (SDLN), long day with low nitrogen (LDLN), short day with high nitrogen (SDHN), and long day with high nitrogen (LDHN). Each value is the means ± SE (*n* = 5). Different letters above bars denote statistically significant differences between treatment at the *p* < 0.05 level according to Tukey’s test.

Compared with SDLN condition, high N supply increased APX activity by approximately 23.2% in NA5 under short day length. APX, CAT, and SOD activities increased by approximately 32.3, 29.5, and 76.1%, respectively, in NA9 under SDHN treatment compared with under SDLN treatment (**Figures 1A–C**). Although long day length significantly increased the APX and SOD activities of both NA5 and NA9 under low N condition, the increases in APX and SOD in NA5 were significantly higher than those in NA9 under the same condition (**Figures 1A,C**). In addition, significant genotype × photoperiod and genotype × photoperiod × N interactions affected APX, CAT, and SOD activities (**Table 2**).

**Table 2:** Statistical significances of single and interactive effects of genotype, photoperiod regime, and nitrogen levels on physiological and biochemical parameters based on three-way ANOVA.

Parameters	SOD	APX	CAT	$O_{2}^{•–}$	MDA	Proline	AsA	GSH	Soluble Sugar	Soluble Protein
*P*: F_G_	ns	^∗∗∗^	^∗∗∗^	^∗^	^∗∗∗^	^∗∗∗^	^∗∗^	^∗∗∗^	^∗∗∗^	ns
*P*: F_D_	^∗∗∗^	^∗∗∗^	ns	ns	ns	^∗∗^	^∗∗∗^	ns	^∗∗∗^	^∗∗∗^
*P*: F_N_	^∗∗^	^∗∗∗^	^∗∗∗^	^∗∗∗^	^∗∗∗^	^∗∗∗^	^∗∗∗^	^∗∗^	^∗∗∗^	^∗^
*P*: F_G × N_	^∗^	ns	^∗∗∗^	ns	^∗∗∗^	ns	^∗∗^	^∗∗∗^	^∗∗∗^	ns
*P*: F_G × D_	^∗∗∗^	^∗∗∗^	^∗∗∗^	^∗∗^	^∗∗∗^	^∗∗∗^	^∗∗∗^	^∗∗∗^	ns	^∗^
*P*: F_N × D_	ns	^∗∗∗^	^∗^	^∗∗∗^	^∗∗∗^	^∗∗^	^∗∗∗^	^∗^	^∗∗∗^	^∗^
*P*: F_G × N × D_	^∗∗^	^∗∗∗^	^∗∗^	^∗^	ns	^∗∗∗^	^∗∗∗^	^∗∗∗^	^∗∗∗^	^∗∗∗^

*AsA, reduced ascorbate; GSH, reduced glutathione; MDA, malondialdehyde; SOD, superoxide dismutase; CAT, catalase; APX, ascorbate peroxidase. F_*G*_, genotype effect; F_*D*_, photoperiod regime effect; F_*N*_, nitrogen level effect; F_*G* × *N*_, the interactive effect of genotype and nitrogen level; F_*G* × *D*_, the interactive effect of genotype and photoperiod regime; F_*N* × *D*_, the interactive effect of photoperiod regime and nitrogen level; F_*G* × *N* × *D*_, the interactive effect of genotype, nitrogen level and photoperiod regime. Significant values of the factorial analysis (ANOVA) for the effects are denoted as: ns, no significant; ^∗^*p* < 0.05; ^∗∗^*p* < 0.01; ^∗∗∗^*p* < 0.001.*

$O_{2}^{•–}$ generation rate remarkably increased in both NA5 and NA9 under LDHN treatment, whereas NA9 exhibited higher $O_{2}^{•–}$ generation rate than NA5 under the same condition (**Figure 1D**). Compared with those under LDLN condition, the rates of $O_{2}^{•–}$ generation increased by approximately 245.6% in NA5 and 131.2% in NA9 under LDHN (**Figure 1D**). When grown under short photoperiod, high N supply significantly increased $O_{2}^{•–}$ generation rate in NA5 by approximately 31.5% compared with those in individuals under SDLN condition, whereas $O_{2}^{•–}$ generation rate significantly decreased by 33.6% in NA9 under the same condition (**Figure 1D**). $O_{2}^{•–}$ generation rate was significantly affected by genotype × photoperiod and N × photoperiod interactions (**Table 2**).

### Biochemical Index

**FIGURE 2:**
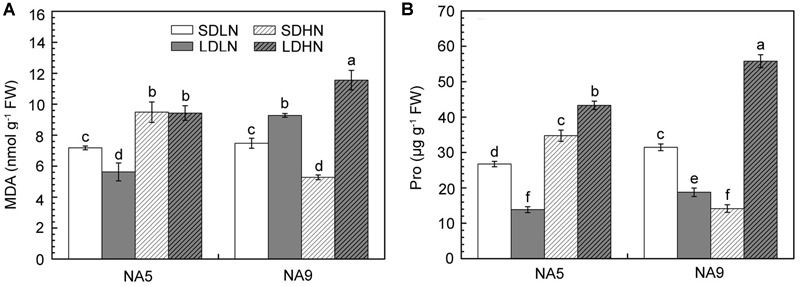
**The content of (A)** malondialdehyde and **(B)** proline in barley plants exposed to the treatments of SDLN, LDLN, SDHN, and LDHN. Each value is the means ± SE (*n* = 5). Different letters above bars denote statistically significant differences between treatment at the *p* < 0.05 level according to Tukey’s test.

**FIGURE 3:**
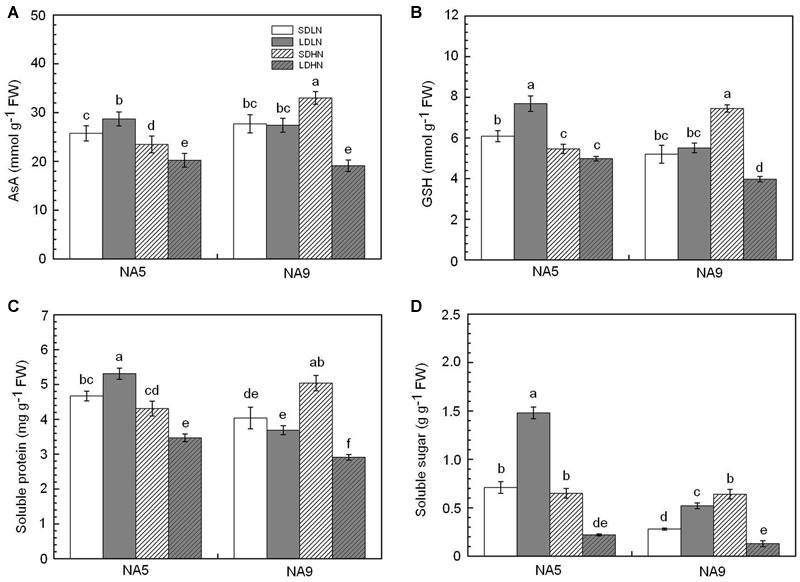
**The content of (A)** reduced ascorbate, **(B)** reduced glutathione, **(C)** soluble protein, and **(D)** soluble sugar in barley plants exposed to the treatments of SDLN, LDLN, SDHN, and LDHN. Each value is the means ± SE (*n* = 5). Different letters above bars denote statistically significant differences between treatment at the *p* < 0.05 level according to Tukey’s test.

When exposed to changes in the external environment, plants develop complex physiological and biochemical metabolism to maintain a stable intracellular status and adapt to new conditions. Irrespective of short or long day lengths, high N supply significantly increased the contents of MDA and Pro in NA5 (**Figure 2**). High N supply increased MDA and Pro by approximately 32.2 and 41.2%, respectively, in NA5. High N supply, however, decreased MDA and Pro by 29.4 and 55.1%, respectively, in NA9 under short day condition compared with those in individuals under SDLN condition (**Figure 2A**). NA9 had significantly higher Pro than NA5 under LDHN treatment; Pro increased by 197.7 and 62.1% in NA9 and NA5, respectively (**Figure 2B**). In addition, high N supply significantly decreased AsA and GSH contents in NA5 under long day condition compared with those in individuals under LDLN condition (**Figure 3**). Compared with other individuals under LDLN condition, GSH content decreased by approximately 35.2 and 21.5% in NA5 and NA9, respectively (**Figure 3B**). Moreover, MDA, Pro, AsA, and GSH contents were significantly affected by genotype × photoperiod and N × photoperiod interactions (**Table 2**).

Under short day condition, high N supply significantly increased soluble protein content in NA9 by approximately 24.7% compared with those in individuals under low N supply (**Figure 3C**). Soluble protein did not significantly change in NA5 under short day condition. High N supply significantly decreased soluble protein by approximately 25.6 and 27.9% in NA5 and NA9, respectively, compared with individuals under long day condition (**Figure 3C**). The soluble sugar in NA9 under high N increased by 128.5% compared with that in NA9 under low N supply when grown under short day condition (**Figure 3D**). Under long day condition, high N supply significantly decreased soluble sugar content in NA5 compared with those in individuals under low N (**Figure 3D**). In addition, both soluble protein and soluble sugar were significantly affected by N × photoperiod and genotype × N × photoperiod interactions (**Table 2**).

### Chloroplast Ultrastructure

**FIGURE 4:**
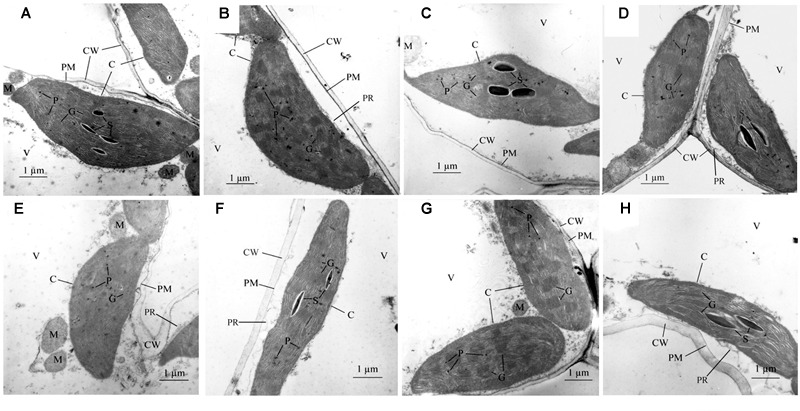
**Electron micrographs of mesophyll chloroplast in barley cultivars of NA5 and NA9 exposed to different treatments. (A)** mesophyll chloroplasts of NA5 under SDLN condition, **(B)** mesophyll chloroplasts of NA5 under LDLN condition, **(C)** mesophyll chloroplasts of NA5 under SDHN condition, **(D)** mesophyll chloroplasts of NA5 under LDHN condition, **(E)** mesophyll chloroplasts of NA9 under LDLN condition, **(F)** mesophyll chloroplasts of NA9 under SDLN condition, **(G)** mesophyll chloroplasts of NA9 under SDHN condition, **(H)** mesophyll chloroplasts of NA9 under LDHN condition. The bars shown are 1 μm. C, chloroplast; CW, cell wall; PM, plasma membrane; P, plastoglobulus; PR, peripheral reticulum; S, starch granule; M, mitochondrion; G, granum; V, vacuole.

The maintained structural integrity of the chloroplast is required for chlorophyll stability and function. Under different treatments, each chloroplast granum was well-developed and densely stacked with 4–25 thylakoids. More plastoglobuli and minor peripheral reticula developed in NA5 under SDHN treatment compared with under SDLN treatment (**Figures 4A,B**). Under LDHN treatment, the thylakoid membranes of NA5 were visibly incomplete, and the number of grana decreased to 3–7 with an average of 15 thylakoids per granum stack (*n* = 30), whereas the number of grana was 18–24 with an average of 22 thylakoids per granum stack (*n* = 30) under LDLN treatment. Moreover, starch accumulation was limited in the mesophyll cells of NA5 under SDLN, SDHN, or LDHN treatment (**Figures 4A,C,D**). More plastoglobuli, visibly discontinuous cell membranes, and rough cell walls were observed in the cytoplasm of NA9 under SDLN treatment than under SDHN treatment (**Figures 4E,G**). The deleterious effects of long photoperiod were more pronounced than those of short photoperiod on NA9, especially in plants under high N supply. In NA9 under LDLN and LDHN treatments, chloroplast shapes varied from typically ellipsoidal, fusiform to irregular, and starch accumulation was visible (**Figures 4F,H**). Furthermore, dilated grana, rough peripheral reticula, and thickened cell walls in chloroplast cells were observed under LDHN treatment (**Figure 4H**).

## Discussion

Plants utilize photoperiod responses to control the timing of flowering during the growing period. The variations in photoperiod responses of many crops enable adaptation to different environments and farming practices. Long photoperiod plays an important role in the promotion of flowering in barley (Turner et al., 2005). Although the literature provides numerous examples of the quantitative responses of cereals to the length of photoperiod transition, little information is available on the responsiveness of N supply to photoperiod length. Previous studies have reported that plants grown under unfavorable long or short photoperiod suffer from serious light-related injuries (Hillman, 1956), whereas increasing N supply relieves the negative effects that are related to biotic and abiotic stress conditions (Ehlting et al., 2007; Yao and Liu, 2007). In the present study, we evaluated the responses of two barley cultivars (NA5 and NA9) with contrasting photoperiod responses under two photoperiod regimes with different N supply conditions.

### Involvement of N in Changes in Photosynthesis and Structural Leaf Traits in Response to Photoperiod Change

Nitrogen supply profoundly influences many aspects of plant growth and development, including root and shoot growth (Tschoep et al., 2009; Lima et al., 2010). In this study, high N supply significantly decreased biomass accumulation, photosynthetic capacity, and pigment content in NA5 but significantly decreased biomass accumulation and photosynthesis in NA9. Chlorophyll is the photosensitizer that generates singlet oxygen in light (Katz et al., 1978). Singlet oxygen participates in chlorophyll degradation during shortened photoperiod (Rosenthal and Camm, 1997). The stability of the *Chl* a/b ratio suggested that *Chl* a and *Chl* a/b-containing complexes are degraded under unfavorable light conditions (Becker et al., 2006). In the present study, the *Chl* a and *Chl* a/b of both NA5 and NA9 significantly increased under LDLN treatment, whereas *Chl* a significantly decreased in NA9 individuals under LDHN treatment (**Table 1**). Some crop plants positively respond to high N supply, whereas others exhibit decreased productivity under high N supply. Thus, plant responses to elevated N remain frustratingly complex (Wallace et al., 2007; He et al., 2010). The results of the present study clearly indicated that long day length and high N supply accelerate chlorophyll degradation, thus decreasing photosynthetic capacity under suboptimal photoperiod conditions.

Stomatal conductance and transpiration rate decreases under environmental stress, which generally limits CO_2_ entry and net photosynthesis (Evans and Loreto, 2000; Medrano et al., 2002). Under short or long photoperiod in NA5 and under long photoperiod in NA9, high N supply considerably decreased *P*_n_, *E, g*_s_, and *C*_i_ relative to low N content. High N supply increased the photosynthetic parameters of NA9 under short photoperiod. Therefore, under high N supply, NA9 is more adaptive to short day length than NA5. These results indicated that high N supply decreases photosynthetic activity, thus negatively affecting barley plants under long photoperiod. The maintained structural integrity of mesophyll cells is required for biological functions under unfavorable environments. The ultrastructure and pigment composition of leaves undergoes a series of changes under sustained environmental shift (Das, 2004). In our experiment, the morphology of mesophyll cells in NA5 was negatively affected by SDHN and LDHN treatments, but only by LDHN in NA9. The control treatment clearly induced a series of morphological changes, such as thickened and distorted cell walls, discontinuous plasma membranes, reduced grana, loosely stacked thylakoids, and vacuolization. In addition, starch accumulated in the chloroplasts of NA5 and NA8 in response to LDHN condition. The excessive accumulation of starch disrupts chloroplast structure, thus decreasing CO_2_ assimilation (Boussadia et al., 2010). These results revealed that short or long day lengths with high N supply adversely affect cell organelles. In NA9, high N content mitigated the damage from oxidative stress and improved acclimation to short day length, as evidenced by the high photosynthetic ability, antioxidant content, antioxidant properties, and more integral chloroplast structure of the cultivar.

Photosynthetic performance is positively correlated with N content because the proteins that are associated with the Calvin cycle and thylakoids represent the majority of leaf N content (Evans, 1989). Rubisco and thylakoid membrane protein account for the majority of soluble protein in leaves (Jiang et al., 1999). Given that N supply and both rubisco and chlorophyll are strongly and linearly correlated, there is a strong causal correlation between soluble protein and photosynthesis. Our findings implied that the significant decreases in soluble protein in each barley cultivar under LDHN condition contribute to negative effects on photosynthesis and thereby reduce biomass, photosynthetic parameters, and pigment content (**Table 1**). Consequently, this result suggested that high N content increases sensitivity to long days in barley by reducing photosynthetic capacity.

### Function of N in the Response of the Antioxidant Defense System to Photoperiod Change

Malondialdehyde is an important marker of membrane lipid peroxidation and reflects oxidative damage to the cell membrane (Gawel et al., 2004). Pro, an amino acid, plays a highly beneficial role in plants and contributes to stabilizing subcellular structures, scavenging free radicals, and buffering cellular redox potential under various stress conditions (Ashraf and Foolad, 2007; Verbruggen and Hermans, 2008). In plants under short day length, increased MDA and Pro induce leaf senescence when antioxidant enzyme activities decrease (Zhao et al., 2009). In this study, we found that LDHN significantly increased MDA and Pro contents in both NA5 and NA9; however, NA9 showed higher MDA and Pro contents than NA5 under long day length with either low N or high N supply. These results indicated that NA9 suffered from more oxidative stress exposure under long day treatment. Moreover, high N supply significantly decreased MDA and Pro content in NA5 and NA9, except for in NA9 under SDHN treatment. NA5 and NA9 possess different acclimations to short or long photoperiod and N supply. Correspondingly, long-day cultivars suffered from the negative effect of high N content on antioxidant capacity; however, for short-day cultivars, N supply might compensate for insufficient daytime exposure and thus shows a promoting effect on growth. In the facultative long-day plant *Arabidopsis*, low N content increases the amplitudes of all the circadian transcripts of LATE ELONGATED HYPOCOTYL, CIRCADIAN CLOCK ASSOCIATED 1, and TIMING OF CAB EXPRESSION 1 throughout the circadian cycle, whereas the high N content reduced the amplitudes of these genes without phase shift (Yuan et al., 2016). Therefore, the molecular regulatory mechanism of the different photoperiod responses of different barley cultivars should be explored.

Shortened day length decreases a series of antioxidant enzyme activities but increases $O_{2}^{•–}$ generation rate (Del Río et al., 2002; Zhao et al., 2009). Previous works have proposed that supplemental N increases the sensitivity of plants to enhanced UV-B (Yao and Liu, 2007) or decreases sensitivity to water and heavy metal stress by altering the anti-oxidative defense system and cell membrane stability (Saneoka et al., 2004; Chen et al., 2011). LDLN treatment significantly increased APX, CAT, and SOD activities in NA5, whereas SDHN treatment increased these antioxidant enzyme activities in NA9. Accordingly, the rate of $O_{2}^{•–}$ generation significantly decreased in NA5 under LDLN treatment and in NA9 under SDHN treatment. High N content generally increased $O_{2}^{•–}$ generation rate in NA5; however, NA9 exhibited higher $O_{2}^{•–}$ generation rate than NA5 under LDHN treatment. N supply affects responsiveness to photoperiod change in barley by changing antioxidant capacity, which confirmed the conclusion that day length influences oxidative stress responses and that ROS is generated and accumulates in the chloroplast in response to photoperiod change (Vollsnes et al., 2009). Changes in AsA and GSH contents are valuable stress indicators in plants and determine the fate of the plant during oxidative stress (Foyer and Noctor, 2011; Heyneke et al., 2013). AsA and GSH contents significantly increased in NA5 under LDLN treatment and in NA9 under SDHN treatment, indicating that the barley plant greatly suffers from lower oxidative stress and has better adaptation under controlled treatments. The decrease in AsA or GSH content implied the inefficient elimination of ROS in NA5 under SDHN and LDHN treatments and in NA9 under LDHN treatment. Soluble sugar detoxifies ROS in chloroplasts and vacuoles by enhancing the production of NADPH, a major cofactor of the ROS scavenging pathway of the ascorbate–glutathione cycles (Couée et al., 2006; Michael et al., 2011). Different day-lengths and light intensities may change soluble sugar concentration in plant tissues, thus modifying plant growth and development (Wingler et al., 2006). Our results showed that sugar content decreased in NA5 and NA9 under LDHN treatment, thereby indicating higher ROS accumulation and oxidative damage. Therefore, high N supply facilitates responsiveness, which indicates reduced anti-oxidative capacity, to photoperiod changes in the barley plant. This finding supported the view that higher N rates sustain adverse effects under stress condition (Pell et al., 1990).

## Author Contributions

JZ designed the research. HS, YL, and YaW performed the research. HS, YiW, HK, XF, LS, and SY analyzed the data and finally JZ and YZ wrote the article.

## Conflict of Interest Statement

The authors declare that the research was conducted in the absence of any commercial or financial relationships that could be construed as a potential conflict of interest. The reviewer DN and handling Editor declared their shared affiliation, and the handling Editor states that the process nevertheless met the standards of a fair and objective review.
